# Master of the Masquerade: An Atypical Presentation of Acute Aortic Dissection

**DOI:** 10.1155/2020/5743985

**Published:** 2020-02-20

**Authors:** Ramy Mando, Daniel Tim, Anthony DeCicco, Justin Trivax, Ivan Hanson

**Affiliations:** ^1^Department of Internal Medicine, Beaumont Health System, Royal Oak, MI, USA; ^2^Department of Cardiovascular Medicine, Beaumont Health System, Royal Oak, MI, USA; ^3^Oakland University William Beaumont School of Medicine, Auburn Hills, MI, USA

## Abstract

Acute aortic dissection (AAD) is associated with unacceptably high mortality rate. As such, early diagnosis and aggressive management are essential in order to avoid life-threatening complications. Herein, we report an atypical presentation of AAD and clinical sequelae.

## 1. Introduction

The incidence of AAD is ~2.3 per 100,000 person-years with mortality rates greater than 50% within 30 days of diagnosis [1]. Furthermore, for every hour that AAD involving the ascending aorta (Sanford type A) is left untreated, mortality rate increases by 1% [2–4]. It is for these reasons that this devastating condition requires a high index of suspicion to make the diagnosis and implement treatment strategies as quickly as possible.

The International Registry of Aortic Dissection (IRAD) was established in 1996 to raise awareness and provide guidance in the diagnosis and treatment of this condition. Overall, type A dissection constitutes 67% of AAD. The most common risk factors for AAD include hypertension (76.6%), atherosclerotic disease (27%), prior cardiac surgery (16%), and Marfan syndrome (5%) [5]. A study utilizing the IRAD with a focus on younger patients (<40 years of age) revealed that this population constituted 7% of patients who suffered AAD. Furthermore, the study identified unique risk factors in this population including Marfan syndrome, bicuspid aortic valves, and larger aortic dimensions [6].

Abrupt and severe chest and back pain are the most common presenting signs and symptoms for type A (79% vs. 43%, respectively) and type B (63% vs. 64%, respectively) aortic dissection. In general, anterior chest pain is more consistent with type A dissection while back pain was found to be more consistent with type B dissection [7]. Acute abdominal pain was the main presenting symptoms in only 4.6% of cases in the IRAD and was associated with increased mortality [8].

Herein, we present a case of AAD that presented with atypical chest pain, abdominal pain, scrotal edema, seizure, pericardial tamponade, and death. Unfortunately, AAD was not suspected until the patient developed refractory cardiac arrest. We hope that our case raises awareness of these atypical features so that future patients may be diagnosed and treated earlier.

## 2. Case Description

**Figure 1:**
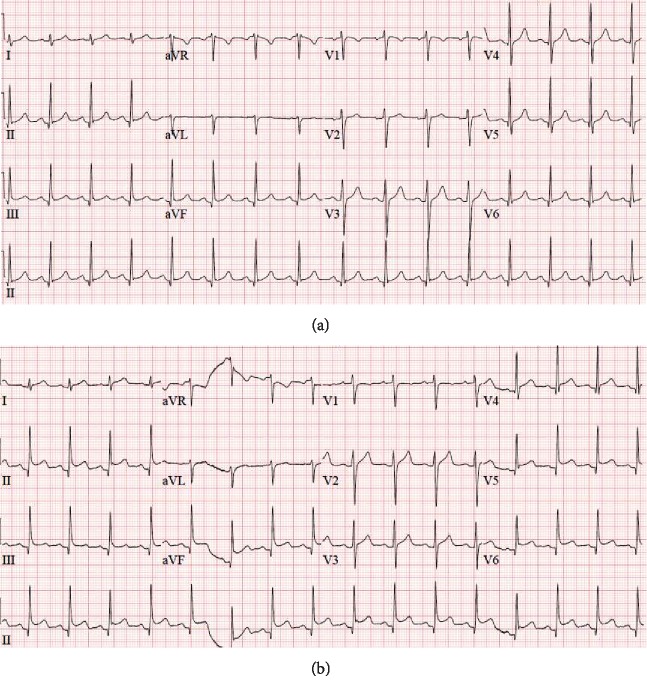
(a) EKG at the initial presentation revealing normal sinus rhythm and no significant ST or T-wave abnormalities. HR 92 bpm. (b) EKG obtained at the time of the second presentation. Notable changes include diffuse ST-elevation. HR 96 bpm.

A 33-year-old male with no past medical history presented to the emergency department (ED) for atypical chest pain described as pleuritic and positional. He described associated nausea, vomiting, diarrhea, epigastric pain, and myalgia. He also reported decreased oral intake for 2-3 days prior to presentation. On examination, blood pressure (BP) was 127/56 mmHg, heart rate (HR) was 94 bpm, and he was afebrile. He was 180 cm tall and weighed 70 kg. He appeared to be in mild distress, exhibited bilateral anterior chest wall and epigastric tenderness. A 12-lead electrocardiogram (ECG) revealed normal sinus rhythm with a rate of 92 bpm, normal axis with no ST or T-wave abnormalities (Figure 1(a)). Initial labs were notable for leukocyte count of 17,200 bil/L, serum creatinine 1.42 mg/dL, and presence of hyaline casts in the urine. Acute abdominal series was unremarkable with the exception of a single mildly dilated small bowel loop consistent with ileus versus enteritis. He was treated with intravenous hydration, antiemetics, morphine, and ketorolac for pain control. On reevaluation, he reported significant symptomatic improvement and was discharged home with close follow-up with his primary care physician. The following day, he presented to the ED with persistent nausea, vomiting, diarrhea, and abdominal pain. He also developed scrotal pain and swelling. Physical examination revealed BP of 117/73 mmHg and HR of 104 bpm. He exhibited continued chest wall tenderness and epigastric pain as well as scrotal swelling and tenderness. 12-lead ECG revealed sinus tachycardia with a rate of 100 bpm, early repolarization changes with ST elevation in the lateral leads (Figure 1(b)). Troponin was mildly elevated at 0.11 ng/mL (elevated from 0.01 ng/mL the day prior). Other labs were notable for an improving leukocytosis (11K bil/L), a new normocytic anemia (12.9 g/dL compared to 14.1 g/dL the day prior), and stable renal function. Liver panel revealed an AST of 33 U/L, ALT of 23 U/L, and total bilirubin of 0.8 mg/dL. Ultrasound of the scrotum revealed right-sided hydrocele and left-sided varicocele. Computed tomography (CT) of the chest, abdomen, and pelvis revealed a moderate pericardial effusion with complex-appearing fluid concerning for hemopericardium. The CT was also notable for periportal edema, perihepatic ascites, bilateral pleural effusion, and scattered free fluid throughout the abdomen and pelvis. He was managed with hydration, morphine, and ketorolac and transferred to our hospital for further management.

During transport, he was noted to have had sudden loss of consciousness associated with clonic jerking, followed by rapid and complete recovery of normal neurologic function. On arrival to the ED, BP was 116/64 mmHg, HR was 96 bpm, and the patient was in no acute distress. Laboratory evaluation was significant for an increase in AST (99 U/L), ALT (101 U/L), and total bilirubin (1.6 mg/dL). A bedside echocardiogram was done and revealed a normal ejection fraction, normal right ventricular diameter, and a moderate pericardial effusion. He was admitted to the inpatient unit with consultations placed to general surgery for abdominal pain and rheumatology for evaluation of serositis. Five hours later, the patient became tachycardic with HR ranging from 140 to 150 bpm and hypotensive with systolic BP around 70 mmHg. Phenylephrine was started immediately with improvement in blood pressure. A bedside echocardiogram revealed a large pericardial effusion, with evidence of diastolic compression of the right ventricle, consistent with pericardial tamponade. He was taken to the catheterization laboratory for emergent pericardiocentesis.

**Figure 2:**
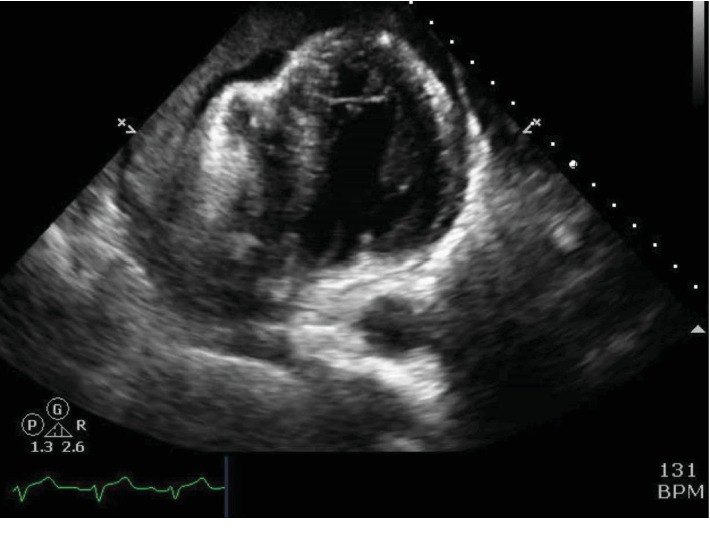
Transesophageal echocardiogram revealing a large pericardial effusion with diastolic compression of the right ventricle.

**Figure 3:**
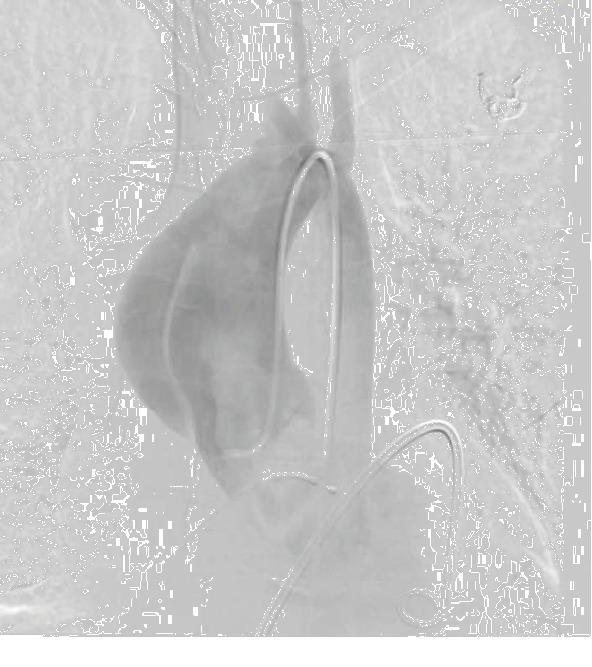
Supravalvular aortography revealing marked dilation of the ascending aorta with a dissection flap beginning just above the right sinus of Valsalva extending to at least the aortic arch.

In the catheterization lab, pericardiocentesis via apical approach was attempted. Agitated saline injection through a microcatheter was performed, but a large part of the pericardial space was occupied by echo-dense material and saline bubbles were not visualized in the pericardial space (Figure 2). Next, subxiphoid approach was attempted. The pericardial space was entered, but again, agitated saline bubbles were not visualized in the pericardial space. However, the wire was successfully passed through the catheter under fluoroscopic guidance, and the position of the wire appeared to be appropriately located in the pericardial space. While attempting to advance a drainage catheter over the wire, the patient had recurrent, sudden loss of consciousness and clonic activity. Subsequently, there was a rapid decline in heart rate and a 12-lead ECG revealed marked sinus bradycardia with inferior ST-elevation. Advanced cardiac life support (ACLS) protocol was initiated including cardiopulmonary resuscitation (CPR), epinephrine/bicarbonate, transvenous pacemaker placement (via right femoral vein), and cardioversion for intermittent ventricular fibrillation. At this point, there was concern for iatrogenic injury of the right coronary artery. Femoral arterial access was obtained, and a right Judkins catheter was advanced into the aortic root. The right coronary artery could not be engaged. A supravalvular aortogram revealed marked dilation of the ascending aorta with a dissection flap beginning just above the sinotubular junction along the outer curve of the ascending aorta, involving the arch and extending to the descending aorta. The right coronary and innominate arteries were occluded (Figure 3). Efforts to stabilize the patient for emergent dissection repair were unsuccessful, and he expired immediately after CPR was stopped. Autopsy revealed extension of aortic dissection from the aortic root to bilateral iliac arteries. The root was dilated, and the aortic valve was tricuspid. Histopathology is pending at the time of this report.

## 3. Discussion

This case is unique and illustrates several points. (1) The presentation of AAD may include atypical chest pain and signs/symptoms of heart failure. (2) Presence of a complex effusion on CT or echocardiogram should raise the possibility of hemopericardium caused by extension of aortic dissection in the appropriate clinical setting. (3) Seizure activity may be a manifestation of AAD due to acute cerebral ischemia.

Patients younger than 40 years of age often do not present with the “typical” risk factors and characteristics [6, 7]. Moreover, the 4.6% patients who present with abdominal pain as their primary complaint have higher mortality rates, likely related to less clinician awareness of this manifestation of AAD. This, unfortunately, may have contributed to the demise of our patient. There are no reports describing nausea, vomiting, and diarrhea as symptoms associated with AAD; however, stimulation of cardiac vagal afferent nerves associated with inferior myocardial infarction may trigger these symptoms [9].

Acute onset testicular swelling and pain was the second atypical feature of AAD. Few cases describing this as the predominant symptom have been published. In one case, a 77-year-old male presented with 2 days of sharp testicular pain. Diagnostic studies were largely unremarkable, and he was admitted for observation. Hours later, he suffered from cardiac arrest and autopsy revealed extensive type A dissection [10]. Another case highlighted a 49-year-old male who presented with sudden onset testicular pain. His physical examination was notable for a significantly reduced BP in the right leg compared to the left leg [11]. In our patient, evidence of scrotal swelling, elevated transaminases, and ascites may have indicated acute right ventricular failure due to ischemic right ventricular dysfunction and/or cardiac tamponade. Alternatively, it is possible that extension of the dissection flap led to acute arterial insufficiency of the mesenteric vasculature.

The presence of enteritis on the initial abdominal X-ray with symptoms of abdominal pain, nausea, vomiting, and diarrhea would be consistent with viral gastroenteritis. The following day, a CT was obtained which revealed a pericardial effusion that “does not measure at simple fluid density,” concerning for hemopericardium. Initially, the patient's symptoms in the first 8 hours were largely attributed to viral pericarditis. However, it should be noted on CT that attenuation values can be obtained with high accuracy. Attenuations greater than those of water are consistent with complex effusions most commonly related to hemopericardium, malignancy, or purulent exudates. As such, CT allows for the differentiation of simple pericardial effusions from chylous, malignant, and hemorrhagic effusions with a high degree of certainty [12, 13]. In this case, despite the misleading symptoms at presentation, our patient should have been immediately referred for a more specific diagnostic examination to further characterize the effusion. Immediate diagnosis and management are critical in the treatment of this catastrophic pathology. The consideration of AAD should be made in patients with evidence of hemopericardium, in the appropriate clinical setting.

The final atypical presentation of AAD manifested in our patient was his seizure-like activity. This was initially observed during his transport to our facility by EMS and prior to his cardiac arrest in the catheterization lab. This is may have been due to global cerebral ischemia in the setting of impending hemodynamic collapse and propagation of the dissection. Neurologic symptoms in the setting of AAD are well described and can be seen in as many as one-third of cases. Symptoms may include seizures, aphasia, unconsciousness, syncope, Horner syndrome, paraparesis, anterior spinal cord syndrome, Brown-Sequard syndrome, ischemic neuropathy, and plexopathy [14].

## 4. Conclusion

AAD is a devastating diagnosis with a high mortality rate that requires a low threshold for making the diagnosis, as earlier intervention leads to reduced mortality. This case illustrates that AAD can masquerade as other, more benign, conditions when signs and symptoms are atypical. We hope this report improves clinician awareness of the less common manifestations of AAD.

## References

[1] Melvinsdottir I. H., Lund S. H., Agnarsson B. A., Sigvaldason K., Gudbjartsson T., Geirsson A. (2016). The incidence and mortality of acute thoracic aortic dissection: results from a whole nation study. *European Journal of Cardio-Thoracic Surgery*.

[2] Coady M. A., Rizzo J. A., Goldstein L. J., Elefteriades J. A. (1999). Natural history, pathogenesis, and etiology of thoracic aortic aneurysms and dissections. *Cardiology Clinics*.

[3] Lindsay J., Hurst J. W. (1967). Clinical features and prognosis in dissecting aneurysm of the aorta. A re-appraisal. *Circulation*.

[4] Tran T. P., Khoynezhad A. (2009). Current management of type B aortic dissection. *Vascular Health and Risk Management*.

[5] Evangelista A., Isselbacher E. M., Bossone E. (2018). Insights from the International Registry of Acute Aortic Dissection: a 20-year experience of collaborative clinical research. *Circulation*.

[6] Januzzi J. L., Isselbacher E. M., Fattori R. (2004). Characterizing the young patient with aortic dissection: results from the International Registry of Aortic Dissection (IRAD). *Journal of the American College of Cardiology*.

[7] Hagan P. G., Nienaber C. A., Isselbacher E. M. (2000). The International Registry of Acute Aortic Dissection (IRAD): new insights into an old disease. *JAMA*.

[8] Upchurch G. R., Nienaber C., Fattori R. (2005). Acute aortic dissection presenting with primarily abdominal pain: a rare manifestation of a deadly disease. *Annals of Vascular Surgery*.

[9] Kawasaki T., Akakabe Y., Yamano M. (2009). Vagal enhancement as evidence of residual ischemia after inferior myocardial infarction. *Pacing and Clinical Electrophysiology*.

[10] Chan-Tack K. M. (2000). Aortic dissection presenting as bilateral testicular pain. *The New England Journal of Medicine*.

[11] Guarín-Loaiza G. M., Nocua-Báez L. C., Alfonso-Hernández G. (2016). Bilateral testicular pain as an acute aortic dissection symptom. *Revista de la Facultad de Medicina*.

[12] O'Leary S. M., Williams P. L., Williams M. P. (2010). Imaging the pericardium: appearances on ECG-gated 64-detector row cardiac computed tomography. *The British Journal of Radiology*.

[13] Bogaert J., Francone M. (2013). Pericardial disease: value of CT and MR imaging. *Radiology*.

[14] Gaul C., Dietrich W., Erbguth F. J. (2008). Neurological symptoms in aortic dissection: a challenge for neurologists. *Cerebrovascular Diseases*.

